# Combining HDAC and MEK Inhibitors with Radiation against Glioblastoma-Derived Spheres

**DOI:** 10.3390/cells11050775

**Published:** 2022-02-23

**Authors:** Eno I. Essien, Thomas P. Hofer, Michael J. Atkinson, Nataša Anastasov

**Affiliations:** 1 Institute of Biological and Medical Imaging, Helmholtz Center Munich, German Research Center for Environmental Health, 85764 Neuherberg, Germany; eno.essien@helmholtz-muenchen.de; 2 Institute of Radiation Biology, Helmholtz Center Munich, German Research Center for Environmental Health, 85764 Neuherberg, Germany; 3 Immunoanalytics Research Group Tissue Control of Immunocytes & Core Facility, German Research Center for Environmental Health, 81377 Munich, Germany; hofer@helmholtz-muenchen.de; 4 Chair of Radiation Biology, Technical University of Munich, 80333 Munich, Germany; m.j.atkinson@tum.de

**Keywords:** glioblastoma, glioblastoma-derived spheres, HDAC inhibitor, MEK inhibitor, radiation, combination therapy

## Abstract

Glioblastoma stem-like cells (GSLCs) in glioblastoma limit effective treatment and promote therapeutic resistance and tumor recurrence. Using a combined radiation and drug-screening platform, we tested the combination of a histone deacetylase inhibitor (HDACi) and MAPK/ERK kinase inhibitor (MEKi) with radiation to predict the efficacy against GSLCs. To mimic a stem-like phenotype, glioblastoma-derived spheres were used and treated with a combination of HDACi (MS-275) and MEKi (TAK-733 or trametinib) with 4 Gy irradiation. The sphere-forming ability after the combined radiochemotherapy was investigated using a sphere formation assay, while the expression levels of the GSLC markers (CD44, Nestin and SOX2) after treatment were analyzed using Western blotting and flow cytometry. The combined radiochemotherapy treatment inhibited the sphere formation in both glioblastoma-derived spheres, decreased the expression of the GSLC markers in a cell-line dependent manner and increased the dead cell population. Finally, we showed that the combined treatment with radiation was more effective at reducing the GSLC markers compared to the standard treatment of temozolomide and radiation. These results suggest that combining HDAC and MEK inhibition with radiation may offer a new strategy to improve the treatment of glioblastoma.

## 1. Introduction

Glioblastoma (GB), a grade IV astrocytoma, is one of the most aggressive primary brain tumors. Despite the adoption of a standard therapy combining surgical resection, radiotherapy and chemotherapy with temozolomide (TMZ), the poor prognoses of patients with GB have failed to improve, with a median survival of only 14.6 months [1]. One factor that limits the success of GB therapy is the presence of a sub-population of glioblastoma stem-like cells (GSLCs) within the tumor [2]. These GSLCs possess characteristics of tissue stem cells, including the ability to self-renew and to generate further malignant progeny [3]. GSLCs are considered to be drug and radiation resistant, as well as promote tumor angiogenesis and tumor recurrence, all of which hinder the effective treatment of GB [2,4,5]. A therapeutic strategy that improves the control of GSLCs offers an opportunity to improve treatment outcomes for GB.

One approach against GB may be to inhibit the MAPK/ERK kinase (MEK), situated downstream of the *RAS*–*RAF*–*MEK*–*MAPK* pathway, stimulating the proliferation and survival of GSLCs [6,7]. The MAPK pathway is activated by a series of phosphorylation events that can be targeted through MEK, the downstream activator of MAPK. Trametinib and TAK-733 are small-molecule-selective MEK inhibitors with antitumor activity in cancers, such as gliomas, multiple myeloma, melanoma and triple-negative breast cancer [8,9,10,11,12]. Trametinib has recently been applied in clinical studies of brain tumors, suggesting that it has the ability to cross the blood–brain barrier (BBB) [13,14]. Although these MEK inhibitors (MEKi) were found to have good safety profiles in clinical trials, they only exhibited limited antitumor activity as single agents due to resistance [10,11,15]. Therefore, MEKi in combination with other anti-cancer agents may lead to more effective therapies. For instance, we recently showed that the effect of TAK-733 on reducing the migratory potential of breast cancer cells was enhanced by 4 Gy irradiation [12].

Various studies reported enhanced antitumor activity when combining MEK and histone deacetylases inhibitors (HDACis) [16,17,18,19]. HDACs are enzymes that decrease acetylation and are epigenetic regulators of gene expression that contribute to the pathogenesis of cancers, such as GB. Therefore, HDACis are considered promising therapeutics for cancer treatment [20,21]. For example, it was shown that the HDACis Entinostat (MS-275) and trichostatin A (TSA) inhibited the formation of GB-derived neurospheres and reduced GB xenograft growth [22]. It is also noteworthy that MS-275 demonstrated the ability to cross the BBB in vivo via increased acetylation of histone H3 in brain tissue of syngeneic rats after intratumoral injection [23]. Several other HDACis showed promising results in preclinical studies but few made it to clinical trials due to limited efficacy for GB therapy as a single treatment [24]. However, the combined effect of HDACis with other anticancer agents seems more promising and is being investigated in preclinical and clinical combination studies [24,25,26].

The combination of an HDACi and a MEKi showed promising results in other cancers [16,27,28] but, to date, has not been explored regarding GB. Clinical studies of GB revealed that monotherapy with newly discovered therapeutics failed to improve survival [29]. In addition, tumor heterogeneity, as well as multiple dysregulated pathways, characterizes GB; therefore, a combination treatment strategy was proposed as the most effective approach to improve therapy [30]. Thus, the aim of our study was to investigate the potential effects of combining the HDACi MS-275 and the MEKi TAK-733 or trametinib with radiation using human GB-derived spheres that mimic a stem-like phenotype. A panel of markers (ALDH1A1, CD133, CD44, Nestin and SOX2) that is associated with stemness to drive tumorigenesis was used to measure and predict the effect of this radiochemotherapy approach against GSLCs. The results demonstrated that this multimodal therapeutic strategy is promising and could offer an opportunity to improve the treatment and survival of GB patients.

## 2. Materials and Methods

### 2.1. Growth and Maintenance of Cell Lines

The human GB cell lines U87 and U251 were obtained from Sirion Biotech GmbH (Martinsried, Germany). Both GB cell lines were cultured in high-glucose Dulbecco’s Modified Eagle Medium (DMEM) with GlutaMAX™-I, 4.5 g/L glucose, pyruvate and 10% FCS. The cell lines were maintained under standard incubator conditions at 37 °C in a humidified atmosphere containing 5% CO_2_. In addition, the U87 and U251 cell lines were cultured as spheres (U87-sph and U251-sph) in a serum-free DMEM/F12 high-glucose medium with GlutaMAX™-I, 4.5 g/L glucose and pyruvate to induce a stem-like phenotype. The stem cell supplements were 1× B27 supplement (Gibco Life Technologies, Darmstadt, Germany), 1× N2 supplement (Gibco Life Technologies, Darmstadt, Germany), 1× Penicillin-Streptomycin (Sigma-Aldrich, Steinheim, Germany), 1× D-(+)-Glucose Solution 45% in H_2_O (Sigma-Aldrich, Steinheim, Germany), 20 ng/mL Epidermal Growth Factor (EGF) Human+ (Sigma-Aldrich, Steinheim, Germany), and 20 ng/mL Fibroblast Growth Factor (FGF) Basic Human+ (Sigma-Aldrich, Steinheim, Germany). The glioblastoma-derived spheres were cultured for at least eight passages and the expressions of stem cell markers were analyzed before they were used for experiments. The cell lines were checked for mycoplasma contamination using the MycoAlert Detection Kit (Lonza Group Ltd., Basel, Switzerland), while the cell line authentication was done via genetic profiling using the PowerPlex^®^ 21 System (Eurofins, Ebersberg, Germany).

### 2.2. Web Database Analysis of GB

The GEPIA web server (http://gepia.cancer-pku.cn, accessed on 19 January 2021) [31] was used to obtain the GB stem cell marker expression. Box plots were downloaded to compare the CD44, Nestin and SOX2 expression levels between non-cancerous (207 samples) and GB tumor samples (163 samples) from the TCGA and GTEx databases.

### 2.3. Immunofluorescence Assay

The assay was performed by seeding 4 × 10^5^ cells (U87, U87-sph, U251 and U251-sph) on microscopic slides placed in 4-well chambered plates and left overnight. The cells were fixed the next day in 4% paraformaldehyde (PFA) for 15 min at room temperature, followed by 3 washes with PBS. Permeabilization was done with 0.2% Triton X-100 for 5 min (only for the intracellular staining of ALDH1A1, SOX2 and Nestin). The cells were washed 2 times in PBS and blocked in 1% BSA and 0.15% glycine in PBS for 1 h at room temperature. After blocking, incubation was done overnight at 4 °C with the following antibodies; ALDH1A1 (36671, 1:100; Cell Signalling Technology, Danvers, MA, USA), CD133 (Ab16518, 1:100; Abcam, Cambridge, UK), CD44 (3570s, 1:1000; Cell Signaling Technology, Danvers, MA, USA), SOX2 (3579s, 1:500; Cell Signaling Technology, Danvers, MA, USA) and Nestin (MA1-110, 1:500; Thermo Fisher Scientific, Darmstadt, Germany). The next day, the cells were washed with PBS and incubated with the secondary antibody mix for 1 h at room temperature. The secondary antibodies were Cy3-Goat Anti-Rabbit (A10520, Red, 1:300) and Alexa Flour 488-Goat Anti-Mouse (A11029, Green, 1:200, Life Technologies, Eugene, OR, USA). The cells were washed 3 times with PBS and cell nuclei were stained with DAPI. Imaging of cells was performed at a magnification of 40× using a Keyence BZ 9000 fluorescence microscope (Keyence, Frankfurt, Germany).

### 2.4. Treatment with HDACi, MEKi and Radiation

The GB-derived spheres were treated according to previously published protocols [26,32]. Briefly, the following conditions were applied: (1) 1 µM of the HDACi MS-275 (S1053; purchased from Selleck Chemicals), (2) 1 µM of the MEKi TAK-733 (S2617; purchased from Selleck Chemicals), (3) 1 µM trametinib (S2673; purchased from Selleck Chemicals), (4) a combination of 1 µM MS-275 plus 1 µM TAK-733 or (5) a combination of 1 µM MS-275 plus 1 µM trametinib. Where specified, the GB-derived spheres were treated with the standard compound TMZ at 50 µM (SC-203292; purchased from Santa Cruz Biotechnology, Dallas, TX, USA) for comparison. All compounds were diluted to give a final concentration of 1% *v*/*v* DMSO and the controls were treated with 1% *v*/*v* DMSO. The GB-derived spheres were treated 72 h after seeding the cells to allow time for sphere formation. After 24 h of compound treatment, the spheres were irradiated at room temperature with X-rays using an X-Strahl RS225 radiation device (X-Strahl LTD, Camberlay, UK). The 4 Gy irradiation dose was delivered at a rate of 0.824 Gy/min using a 3 mm aluminum filter. The sham irradiated controls were handled under the same conditions but were not exposed to radiation.

### 2.5. Cell Viability Assay

Cell viability was tested using CellTiter-Glo^®^ Luminescent Cell Viability Assays (Cat.Nr. G75751) according to the manufacturer’s instructions (Promega, Madison, WI, USA) and previously published data [12]. U87-sph and U251-sph cells were seeded at 3 × 10^5^ cells per well in 12-well ultra-low-attachment (ULA) plates (Corning, NY, USA). Since the spheres were dissociated into single cells for seeding, the spheroid formation was allowed for 72 h. This was followed by treatment with increasing concentrations of MS-275, TAK-733, trametinib and TMZ at 1 µM, 10 µM and 50 µM. Irradiation was done 24 h after treatment at 4 Gy and incubated for an additional 72 h to have a final time point for analysis of 96 h. After this period, the spheres were dissociated with Accutase and counted to re-seed them at 1 × 10^4^ in 96-well ULA plates. The cell CellTiter-Glo^®^ reagent was added after 72 h under cell culture conditions. Incubation was done for 10 min at room temperature before recording the luminescence at 560 nm emission using an infinite M200 plate reader (TECAN, Maennedorf, Switzerland). The measurements were performed in quadruplicates for three independent experiments.

### 2.6. Sphere Formation Assay

After 72 h of treatment (described in Section 2.4) with both compounds and radiation, the GB-derived spheres were harvested and reseeded in triplicates at 200 cells per well in 96-well ULA plates (Corning Inc., Corning, NY, USA) and cultured for at least 2 weeks. The images of spheres in each well were taken using an Operetta imaging system (PerkinElmer, Waltham, MA, USA). The images were taken using the brightfield channel and 10× magnification, while the sphere number per well was counted manually. The sphere formation rate was determined by the number of spheres formed divided by the total number of starting cells.

### 2.7. Western Blot Analysis

Cell pellets were collected after 72 h of compound and radiation treatment (described in Section 2.4). Lysing, protein extraction and immunoblotting were performed as previously described [12]. The target proteins of the GSLC markers were detected with the same antibodies used for immunofluorescence staining listed in Section 2.3, including Acetyl-Histone H3 (9677) and Histone H3 (4499, 1:1000; Cell Signaling, Danvers, MA, USA), MAPK (9101) and phospho-MAPK (9102, Cell Signaling, Danvers, MA, USA), with β-Actin (A5441, 1:20,000; Sigma Aldrich, Steinheim, Germany) as the loading control. The secondary antibodies were horseradish peroxidase-conjugated anti-mouse (A16066, 1:20,000) and anti-rabbit (A16096, 1:10,000; Invitrogen, Carlsbad, CA, USA). The bands of the secondary bound antibodies were detected using enhanced chemiluminescence (ECL) (Amersham, England) reagents. The luminescent signal was detected and captured using an Alpha Innotech ChemiImager system (Biozym, Hessisch Oldendorf, Germany). The GSLC markers ALDH1A1 and CD133 were not detected well using Western blotting and, therefore, excluded from further analysis.

In the case of reprobing, the membranes were stripped with Restore PLUS Western Blot Stripping Buffer (Thermo Scientific, Rockford, IL, USA) for 15 min at room temperature. For the quantification of the band intensities, the Image-J image analysis software [33] was used.

### 2.8. Flow Cytometry Analysis

The cells were harvested 72 h after treatment (described in Section 2.4) and washed once with PBS. Afterward, the cells were blocked in Anti-Hu Fc Receptor Binding Inhibitor (14916173, 1:10 in PBS, Invitrogen, Carlsbad, CA, USA) for 10 min at 4 °C and then washed with PBS. Live–dead staining of cells was done via incubation with a Zombie Aqua™ Fixable Viability Kit (423101, 1:100 in PBS, BioLegend, San Diego, CA, USA) for 30 min at room temperature. After washing in FACS buffer (0.5% BSA in PBS), the cells were stained with BV-785-conjugated CD44 (103041, 1:100; Biolegend, San Diego, CA, USA) diluted in FACS buffer and incubated for 30 min at 4 °C. Next, the cells were fixed in 1× fixation buffer for 30 min at room temperature and washed with 1× permeabilization buffer using the Foxp3/Transcription Factor Staining Buffer Set (5523, Invitrogen, Carlsbad, CA, USA). This was followed by incubation with an antibody mix of APC-Conjugated Nestin (MA5-23650, 1:100; Thermo Fisher Scientific, Darmstadt, Germany) and PerCP-Cy5.5-Conjugated SOX2 (561506, 1:50; BD Biosciences, San Diego CA, USA) in 1× permeabilization buffer for 1 h at room temperature. Additionally, staining with PerCP-Cy™5.5 Mouse IgG1 κ Isotype Control (550795, 1:50; BD Biosciences, San Diego CA, USA) was applied. The cells were washed twice in 1× permeabilization buffer, resuspended in PBS and passed through a 40 µm mesh filter into FACS tubes to remove clumped cells and obtain a single-cell suspension.

The cells were analyzed via flow cytometry using a CytoFLEX LX Flow Cytometer and CytExpert software (Beckman Coulter, Krefeld, Germany). The CytoFLEX LX instrument has a capacity for 21 fluorescence detections and is equipped with a 355 nm (UV) laser, 405 nm (violet) laser, 488 nm (blue) laser, 561 nm (yellow-green) laser, 638 nm (red) laser and 808 nm (infrared) laser. Fluorescence and side scatter light of the CytoFLEX LX were delivered via fiber optics to avalanche photodiode detector arrays, while the emission profiles were collected using reflective optics and single-transmission band-pass filters. Unstained cells were used to set the voltages, while compensation beads (BD Biosciences, San Diego, CA, USA) were used for compensation to correct for spectral overlap across the fluorescent channels. The gating strategy to set a cut-off for negative and positive populations was done using two gating controls. First, unstained cells were used to set negative and positive gates, while the fluorescence minus one (FMO) control was used to address any spillover-induced background [34]. Additionally, an isotype control for SOX2 was included to set gates against non-specific antibody binding. The gating region on the controls was set to contain less than 1% of the cells for both single- and double-positive populations (Figures S4, S5 and S7).

### 2.9. Statistical Analysis

All experiments consisted of three biological replicates unless otherwise indicated and the data represent the mean ± standard error of the mean (SEM). The differences in mean values between two groups (control and treated) were compared using Student’s t-tests and statistical significance defined with *p*-values as follows: * *p* ≤ 0.05, ** *p* ≤ 0.01 and *** *p* ≤ 0.001.

## 3. Results

### 3.1. Influence of Radiation Alone on Specific GSLC Marker Expression

**Figure 1:**
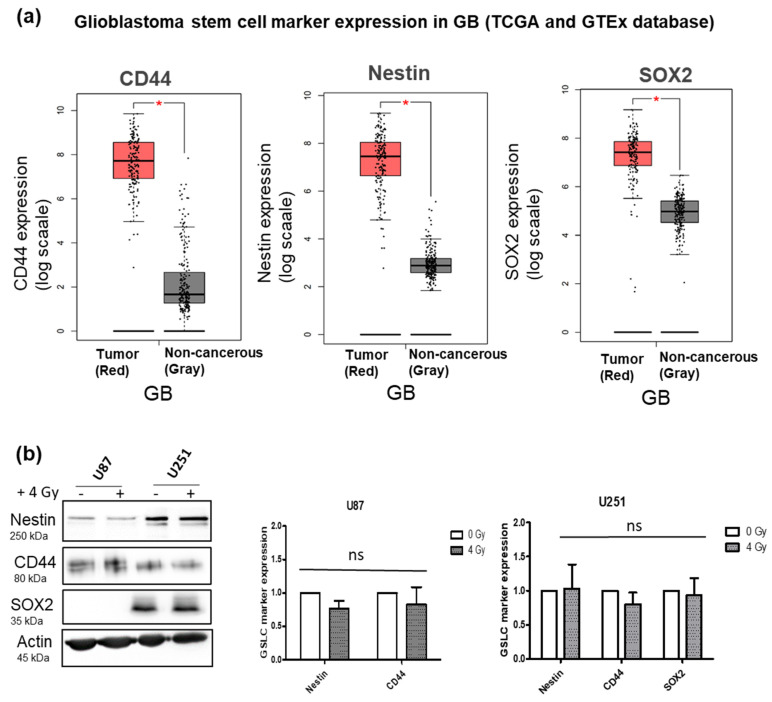
Glioblastoma stem cell marker expression in GB (from TCGA and GTEx database) and GB cell lines: (**a**) TCGA database comparison of GB stem cell marker (CD44, Nestin and SOX2) expression between GB (red; 163 samples) and non-cancerous tissue (gray; 207 samples). Box plots derived from matching TCGA normal and GTEx data downloaded via the GEPIA webserver (* *p*-value < 0.05). (**b**) Protein expression of CD44, Nestin and SOX2 in U87 and U251 GB cell lines and the effect of 4 Gy irradiation. Data represent mean values of three replicates and the error bars ± SEM (*n* = 3; ns—nonsignificant).

The expression of CD44, Nestin and SOX2 in the GB samples and adjacent non-cancerous brain tissue were examined using the GEPIA webserver to interrogate publicly available gene expression databases from the TCGA and GTEx projects. Within the matched TCGA normal and GTEx data, 163 GB tumor samples and 207 non-cancerous samples were analyzed. The gene expression of CD44, Nestin and SOX2 were all significantly higher in GB tumor samples than in the non-cancerous tissue samples (Figure 1a).

**Figure 2:**
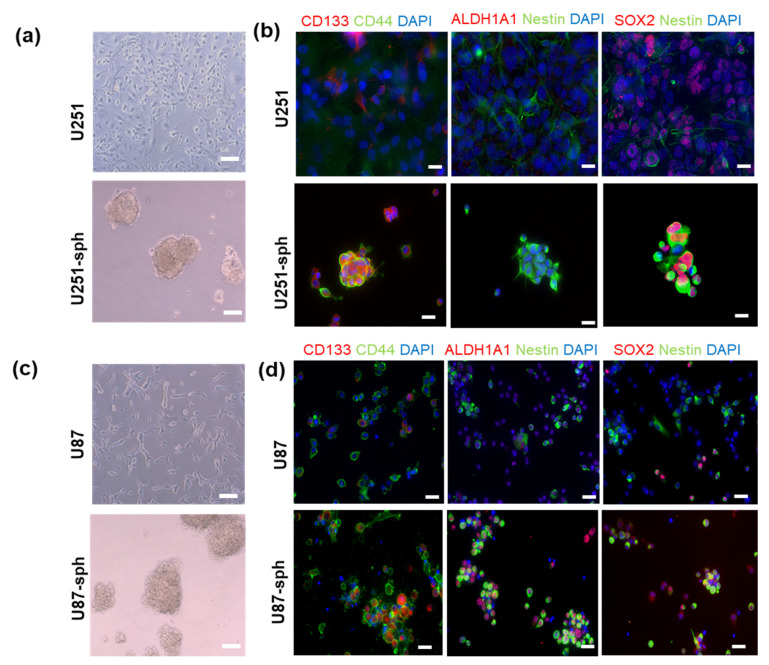
Induction of the stem-like phenotype in U251 and U87 cell lines by a spheroid culture. (**a**) Representative images of the morphology of U251 (cultured in the medium containing 10% FCS) and U251-sph (cultured in the serum-free medium; scale bar: 100 µm). (**b**) Representative images of co-immunofluorescence staining of U251 and U251-sph. The U251-sph cells showed an increased dual expression of the GSLC markers CD133 (red), CD44 (green), Nestin (green) and SOX2 (red) compared to the U251 cells. Nuclei were stained with DAPI (blue). Scale bar: 100 µm. (**c**) Representative images of the morphology of U87 (cultured in the medium containing 10% FCS) and U87-sph (cultured in the serum-free medium; scale bar: 100 µm). (**d**) Representative images of immunofluorescence staining of U87 and U87-sph. The U87-sph cells showed an increased co-expression of the GSLC markers CD133 (red), CD44 (green), ALDH1A1 (red), Nestin (green) and SOX2 (red) compared to the U87 cells. Nuclei were stained with DAPI (blue). Scale bar: 100 µm.

The protein expressions of CD44, Nestin and SOX2 were further detected in the U87 and U251 human GB cell lines. All three markers could be detected in both cell lines, except for SOX2 not detected in U87. Additionally, the effect of radiation alone was investigated and it was observed that there was no beneficial effect on the expression of the GSLC markers 72 h after 4 Gy radiation in vitro (Figure 2b). Since Nestin, CD44 and SOX2 are associated with stemness in GB, their elevated levels in the GB tumor samples suggested that these markers may drive the progression and radioresistance of GB.

### 3.2. Induced GSLC Marker Expression by GB-Derived Spheroid Culture in Serum-Free Medium

To determine whether a stem-like phenotype was induced by the spheroid culture in the serum-free medium in vitro, co-immunofluorescence staining of GSLC markers was performed. After eight passages in the serum-free medium, the GB-derived spheres and their parental cell lines were immunostained to compare the GSLC marker levels in both the serum-free and serum-containing culture conditions. The results showed an increased co-expression of CD133 and CD44, ALDH1A1 and Nestin or SOX2 and Nestin in U87-sph cells (grown in serum-free medium) compared to U87 cells (grown in medium containing 10% FCS) (Figure 2b). Most of the U251-sph cells expressed CD133, CD44, Nestin and SOX2, while fewer U251 cells expressed these stem cell markers. However, ALDH1A1 was not detected in both. Similar results were observed in the U87 parental cells and U87-sph cells. More cells expressed all GSLC markers in U87-sph compared to U87 cells (Figure 2d). These results implied that the culture of the GB cell lines in the serum-free medium could enrich GSLC marker expression.

### 3.3. HDAC and MEK Inhibitors with 4 Gy Radiation Decreased Cell Viability and Sphere Formation

To identify the most potent concentration of the inhibitors in combination with 4 Gy radiation, U87-sph and U251-sph were treated in an increasing concentration range of 1, 10 and 50 µM and the cell viability was determined 72 h after radiation exposure. For both U251-sph and U87-sph, the viability was significantly decreased by the HDACi (MS-275) or the MEKi (TAK-733 or trametinib) alone and with 4 Gy irradiation in a concentration-dependent manner (Figure S2a–f). Interestingly, the viability of the U251-sph cells was significantly increased by treatment with the standard compound TMZ, even at 50 µM; when combined with radiation, no change was observed in comparison to the 4 Gy irradiation alone (Figure S2d–f). This suggested that the HDAC and MEK inhibitors were more potent at lower concentrations (1 µM) compared to the standard compound TMZ (50 µM) used for radiochemotherapy of GB.

The activities of the inhibitors were additionally validated in both GB-derived spheres. Treatment with MS-275 at 1 and 10 µM increased the amount of acetylated histone H3, indicating that HDACs were inhibited (Figure S1a). The inhibitory effects of TAK-733 and trametinib at 1 and 10 µM were confirmed by low amounts of activated MAPK (pMAPK) compared to MAPK (Figure S1b).

**Figure 3:**
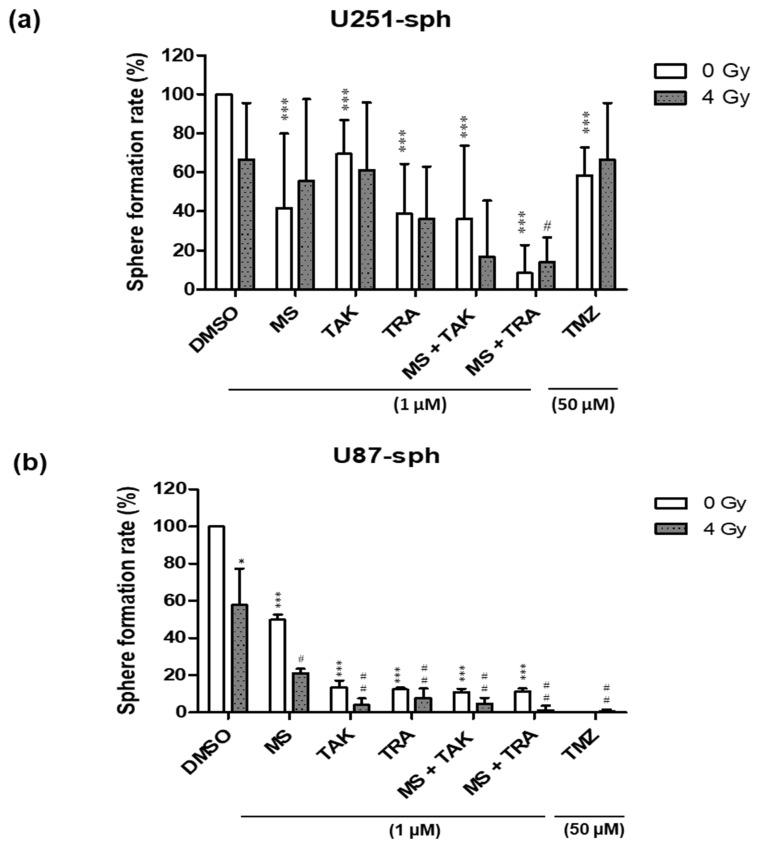
Treatment of HDAC and MEK inhibitors with radiation-inhibited sphere formation of GB-derived spheres. (**a**) Quantification of sphere formation rate (%) after the treatment of U251-sph and (**b**) U87-sph with HDCAi (1 µM MS-275 (MS)), MEKi (1 µM TAK-733 (TAK) or 1 µM trametinib (TRA)), a combination of both (1 µM MS-275 + 1 µM TAK-733 or 1 µM MS-275 + 1 µM trametinib) and 50 µM TMZ alone or with 4 Gy radiation. The sphere formation rate was normalized to the DMSO-treated cells as the control, which were set to 100%. Data represent the mean of 3 independent experiments performed in triplicates (*n* = 3; ± SEM). Asterisks indicate significant differences between 0 Gy DMSO and treated samples using Student’s *t*-test: * *p* ≤ 0.05 and *** *p* ≤ 0.001, while hash symbols indicate significant differences between 4 Gy DMSO and 4 Gy treated samples using Student’s *t*-test: ^#^ *p* ≤ 0.05 and ^##^ *p* ≤ 0.01.

To further investigate the effect of combining the inhibitors with radiation compared to either alone, the sphere-forming ability of U87-sph and U251-sph were also tested after treatment. Radiation alone (4 Gy) reduced the number of spheres formed in U87-sph, but not significantly in U251-sph, while the HDACi and MEKi alone at 1 µM significantly reduced the number of spheres formed in both (Figure 3a,b and Figure S3). The effect of the inhibitors alone was significantly enhanced in U87-sph when combined with radiation, but not significantly in U251-sph. Additionally, the combination of the HDACi and MEKi (MS-275 and TAK-733 or MS-275 and trametinib) alone at 1 µM further significantly reduced the number of spheres formed in both U251-sph and U87-sph compared to the control (*p* ≤ 0.001) (Figure 3a,b). Upon the addition of 4 Gy radiation, the combined inhibitory effect was more enhanced in U87-sph (*p* ≤ 0.01) compared to U251-sph (*p* ≤ 0.05) (Figure 3a,b). Treatment with the standard compound TMZ (at 50 µM) alone or with radiation was more effective in reducing sphere formation in the U87-sph cells compared to the U251-sph cells (Figure 3a,b). Since sphere formation measures the self-renewal of stem-like cells [35,36,37], these results suggest that the combined treatment of the HDACi and MEKi with radiation could potentially decrease the self-renewal ability of GSLCs.

### 3.4. Differential Responses of GSLC Marker Protein Levels to the Combination of HDACi and MEKi with Radiation

In order to determine whether the combination of the HDAC and MEK inhibitors with radiation was effective against the GSLC marker (Nestin, CD44 and SOX2) protein levels, Western blot quantification was performed 72 h after the combined treatment.

**Figure 4:**
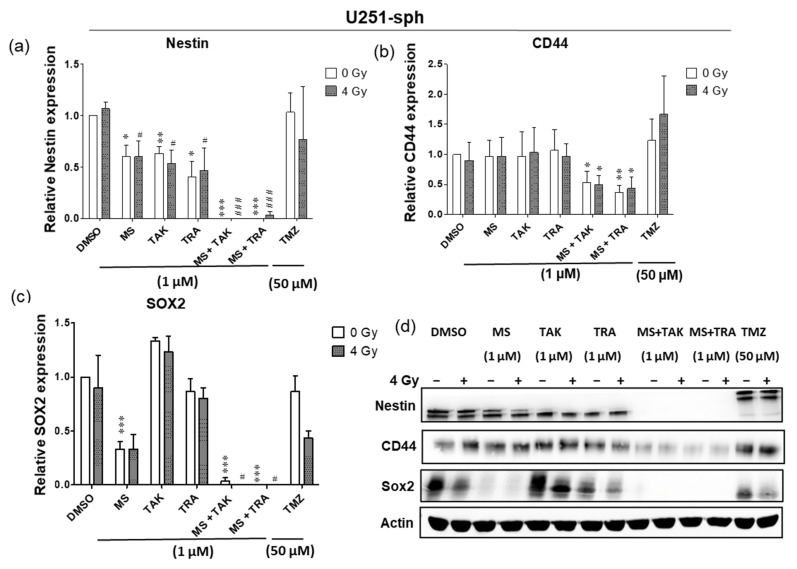

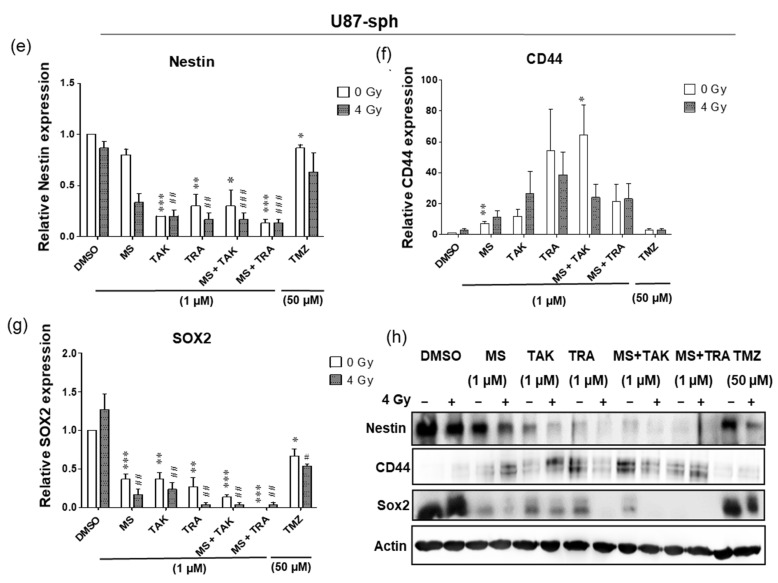
Differential responses of GB-derived spheres to the combination of HDAC and MEK inhibition with radiation. (**a**) U251-sph protein levels of Nestin, (**b**) CD44 and (**c**) SOX2 72 h after the spheres were treated with HDCAi (1 µM MS-275), MEKi (1 µM TAK-733 or 1 µM trametinib), a combination of both (1 µM MS-275 + 1 µM TAK-733 or 1 µM MS-275 + 1 µM trametinib) and 50 µM TMZ alone or with 4 Gy radiation. (**d**) Representative Western blots of GSLC marker protein levels in U251-sph. (**e**) U87-sph protein levels of Nestin. (**f**) CD44 and (**g**) SOX2 after the GSLCs were treated with HDCAi (1 µM MS-275), MEKi (1 µM TAK-733 or 1 µM trametinib), a combination of both (1 µM MS-275 + 1 µM TAK-733 or 1 µM MS-275 + 1 µM trametinib) and 50 µM TMZ alone or with 4 Gy radiation. (**h**) Representative Western blots of GSLC marker protein levels in U87-sph. Relative GSLC marker expressions were first normalized to Actin and then to the sham irradiated control cells treated with DMSO. *n* = 3; ± SEM. Asterisks indicate significant differences between 0 Gy DMSO and treated samples using Student’s *t*-test: * *p* ≤ 0.05, ** *p* ≤ 0.01 and *** *p* ≤ 0.001, while hash symbols indicate significant differences between 4 Gy DMSO and 4 Gy treated samples using Student’s *t*-test: ^#^ *p* ≤ 0.05, ^##^ *p* ≤ 0.01 and ^###^ *p* ≤ 0.001.

In U251-sph, radiation alone did not change the protein level of all markers; however, treatment with the HDACi or MEKi alone and with radiation reduced Nestin (Figure 4a). A decrease in SOX2 via treatment with the HDACi MS-275 alone or with radiation was detected, while CD44 was not changed by the compounds alone or with radiation (Figure 4b). Subsequently, the combination of the HDACi and MEKi (MS-275 and TAK-733 or MS-275 and trametinib) alone or with radiation significantly eradicated Nestin (*p* ≤ 0.001) and SOX2 (*p* ≤ 0.05, *p* ≤ 0.001) and significantly reduced CD44 (*p* ≤ 0.05, *p* ≤ 0.01) (Figure 4a–d).

Comparable results were detected in U87-sph, where treatments with either HDACi or MEKi alone and with radiation significantly reduced the protein level of Nestin and SOX2 (Figure 4e,g). Upon combination of both HDACi and MEKi with radiation, the protein level of SOX2 was significantly eradicated (*p* ≤ 0.01), while Nestin remained significantly decreased (*p* ≤ 0.001) (Figure 4e,g,h). Contrary to U251-sph, the CD44 protein level was increased in U87-sph by all the different treatment conditions (Figure 4f). Furthermore, the treatment with the standard compound TMZ alone or with radiation in both cell lines was less effective against the GSLC marker protein levels compared to the combination treatments (Figure 4a–h). Overall, these results suggested that, while combining the HDACi and MEKi with radiation has great potential to reduce the protein levels of GSLC markers, there may be differential effectiveness against CD44 levels.

### 3.5. Single and Double Expression of GSLC Markers Reduced by the Combination of HDACi and MEKi with Radiation

**Figure 5:**
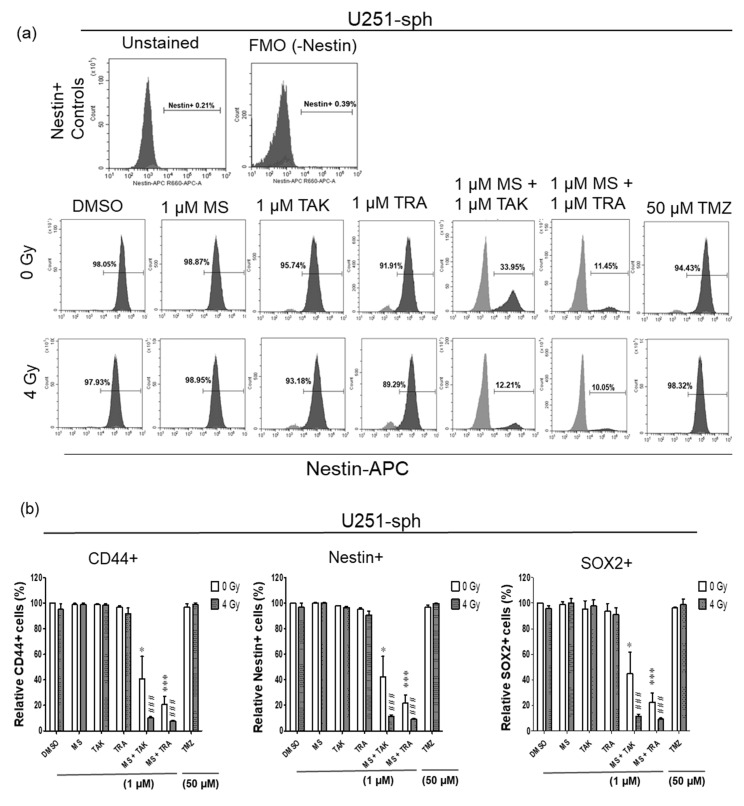

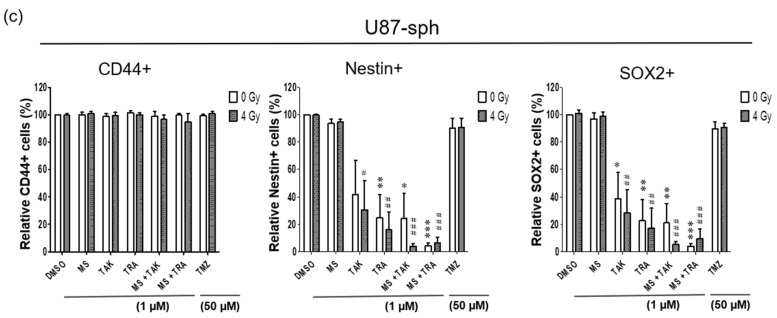
Single expression of GSLC markers reduced by the combination of HDAC and MEK inhibitor with radiation. (**a**) Representative example of flow cytometric histograms of Nestin in U251-sph 72 h after the spheres were treated with HDCAi (1 µM MS-275), MEKi (1 µM TAK-733 or 1 µM trametinib), a combination of both (1 µM MS-275 + 1 µM TAK-733 or 1 µM MS-275 + 1 µM trametinib) and 50 µM TMZ alone or with 4 Gy radiation. Upper histograms represent the unstained sample and fluorescence minus one (FMO) control for the gating percentage of Nestin-positive cells (Nestin+). Lower histograms show the percentage of Nestin-positive cells after the indicated treatment conditions and 4 Gy irradiation. Values inside each histogram represent the percentage of positive single cells from a total of approximately 2 × 10^4^ cells acquired. (**b**) Quantification of CD44+, Nestin+ and SOX2+ relative to sham irradiated control cells (DMSO) set to 100% in U251-sph and (**c**) U87-sph. Data represent means ± SEM (*n* = 3). Asterisks indicate significant differences between 0 Gy DMSO and treated samples using Student’s *t*-test: * *p* ≤ 0.05, ** *p* ≤ 0.01 and *** *p* ≤ 0.001, while hash symbols indicate significant differences between 4 Gy DMSO and 4 Gy treated samples using Student’s *t*-test: ^#^ *p* ≤ 0.05, ^##^ *p* ≤ 0.01 and ^###^ *p* ≤ 0.001.

The effects of the combined inhibitor and radiation treatment were subsequently evaluated using flow cytometry analysis of the GSLC markers. Similar to the protein analysis results, only the combination of the HDACi and MEKi (MS-275 and TAK-733 or MS-275 and trametinib) could significantly reduce the Nestin+, CD44+ and SOX2+ populations in U251-sph (Figure 5a,b). However, these decreases in the positive GSLC marker populations were significantly more pronounced when combined with 4 Gy irradiation (*p* ≤ 0.001) (Figure 5a,b and Figure S5). For example, the Nestin+ population in U251-sph decreased from 97 ± 3% with 4 Gy irradiation alone to 42 ± 12% when treated with MS-275 and TAK-733 alone and a further reduction to 12 ± 1% by adding 4 Gy irradiation to the combination (Figure 5a,b). A similar effect of radiation was also observed with the combination of MS-275 and trametinib in U251-sph (Figure 5a,b).

In U87-sph, the Nestin+ and SOX2+ populations were significantly decreased by the MEK inhibitors (TAK-733 and trametinib) alone and with 4 Gy irradiation (Figure 5c). Upon the combination of the MEK inhibitors with the HDACi MS-275 and 4 Gy irradiation, a significantly stronger decrease was detected (*p* ≤ 0.001) (Figure 5c and Figure S7b,c). However, CD44 expression was not affected by all treatment conditions in U87-sph (Figure 5c and Figure S7a).

**Figure 6:**
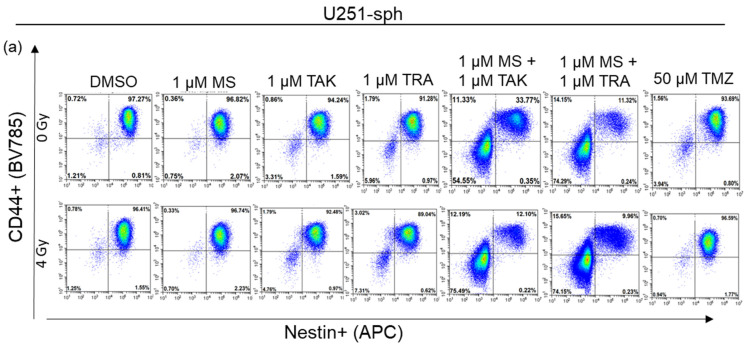

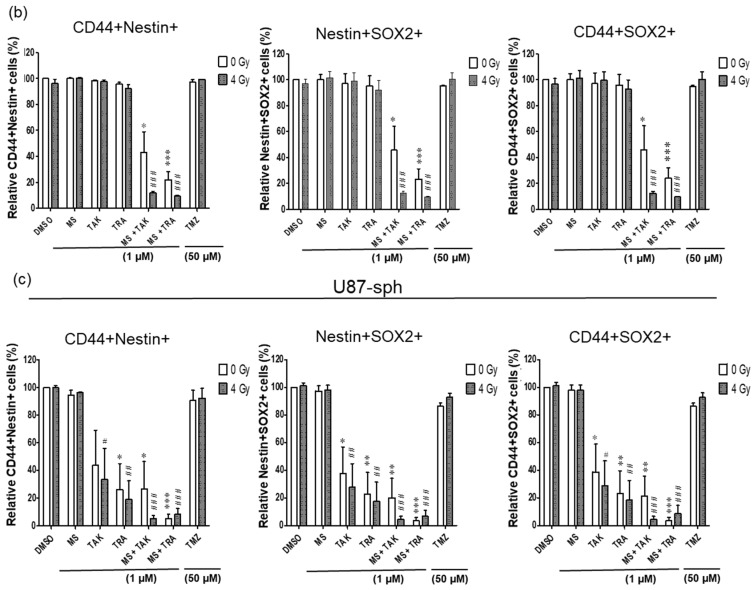
Double expression of GSLC markers reduced by the combination of HDAC and MEK inhibitors with radiation. (**a**) Representative example of flow cytometric plots in U251-sph 72 h after the spheres were treated with HDCAi (1 µM MS-275), MEKi (1 µM TAK-733 or 1µM trametinib), a combination of both (1 µM MS-275 + 1 µM TAK-733 or 1 M MS-275 + 1 µM trametinib) and 50 µM TMZ alone or with 4 Gy radiation. Percentages of double-positive cells for CD44 and Nestin (CD44+Nestin+; upper-right quadrant) after the indicated treatment conditions are shown. Values inside each plot represent the percentage of single cells from a total of approximately 2 × 10^4^ cells acquired. (**b**) Quantification of CD44+Nestin+, Nestin+SOX2+ and CD44+SOX2+ relative to sham-irradiated control cells (DMSO) set to 100% in U251-sph and (**c**) U87-sph. Data represent means ± SEM (*n* = 3). Asterisks indicate significant differences between 0 Gy DMSO and treated samples using Student’s *t*-test: * *p* ≤ 0.05, ** *p* ≤ 0.01 and *** *p* ≤ 0.001, while hash symbols indicate significant differences between 4 Gy DMSO and 4 Gy treated samples using Student’s *t*-test: ^#^ *p* ≤ 0.05, ^##^ *p* ≤ 0.01 and ^###^ *p* ≤ 0.001.

It is now widely accepted that the GSLC population is more accurately identified by the expression of more than one GSLC marker. Since a multicolor approach was used, we further analyzed the co-expression changes of the GSLC markers (CD44+Nestin+, Nestin+SOX2+ and CD44+SOX2+) after the combined treatment with radiation. All three double-positive populations of cells were significantly reduced by the MEK inhibitors (TAK-733 and trametinib) alone or in combination with 4 Gy radiation in U87-sph, but not in U251-sph (Figure 6b,c).

Additionally, combining HDACi and MEKi significantly reduced all double-positive populations in both U251-sph and U87-sph (Figure 6, Figures S6 and S8). However, further exposing the combined inhibitors to 4 Gy radiation significantly enhanced the reduction of all three double-positive populations in both GB-derived spheres (*p* ≤ 0.001) (Figure 6, Figures S6 and S8). This enhanced effect of radiation was more evident in U251-sph than in U87-sph (Figure 6b,c). Again, the standard compound TMZ alone or with 4 Gy radiation was less effective against the single or double expression of the GSLCs markers (Figure 5 and Figure 6). Taken together, these results suggested that the combination of HDACi and MEKi with radiation could reduce the GSLC marker expression more efficiently than the standard treatment of TMZ and radiation.

### 3.6. Population of Dead Cells Increased by the Combination of HDACi and MEKi with Radiation

Live–dead staining was performed during flow cytometry antibody staining using a Zombie Aqua dye that is permeant to dead cells due to compromised membranes and non-permeant to live cells. Therefore, it was possible to access the live versus dead status of the cells after the different treatment conditions.

**Figure 7:**
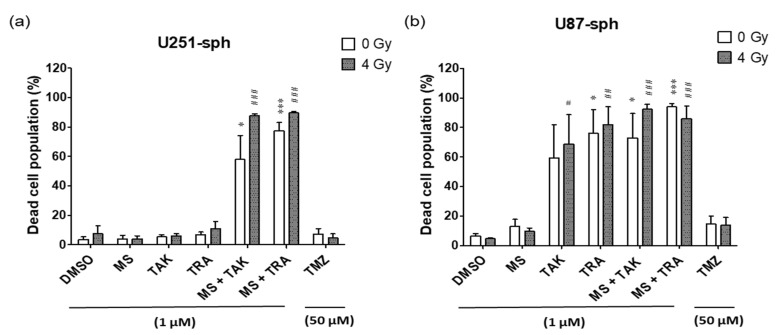
High population of dead cells in GB-derived spheres after treatment with HDACi or MEKi as single or combined compounds with radiation. Quantification data showing the percentage of dead cells in (**a**) U251-sph and (**b**) U87-sph after the different treatment conditions. Data represent mean values ± SEM (*n* = 3). Asterisks indicate significant differences between 0 Gy DMSO and treated samples using Student’s *t*-test: * *p* ≤ 0.05 and *** *p* ≤ 0.001, while hash symbols indicate significant differences between 4 Gy DMSO and 4 Gy treated samples using Student’s *t*-test: ^#^ *p* ≤ 0.05, ^##^ *p* ≤ 0.01 and ^###^ *p* ≤ 0.001.

In U251-sph, the dead cell population was not significantly affected by the single treatments of HDACi or MEKi alone and with radiation (Figure 7a). Upon combination, a significant increase in the percentage of dead cells was detected. The dead cells increased from 8 ± 5% with only 4 Gy radiation to 58 ± 16% with MS-275 and TAK-733 alone and further to 88 ± 1% by adding radiation to the combination. Similarly, the percentage of dead cells was 78 ± 6% with MS-275 and trametinib alone and further increased to 90 ± 1% by including radiation.

Likewise, in U87-sph, the dead cell population was unchanged by single treatments of HDACi and radiation (Figure 7b). Although the MEKi alone and with radiation increased the dead cell population, the highest increase was detected upon the combined treatment of the MEKi and HDACi with radiation.

In contrast, the standard treatment of TMZ and radiation did not significantly affect the dead cell population in both U251-sph and U87-sph.

## 4. Discussion

Radiation plus TMZ remains the most effective non-surgical therapy for GB, although this is mostly palliative due to the radioresistance of GSLCs present within the tumor [2]. Furthermore, GB patients often develop resistance to the DNA-alkylating agent TMZ, along with severe side effects [38]. HDAC and MEK inhibitors have shown promising results as anticancer agents in GB and other tissues [9,12,39]. However, they are considered more potent when combined with other anticancer agents [40,41]. The combination of HDAC and MEK inhibitors as a chemotherapeutic strategy in tumors was first proposed in a study that showed MEK inhibitors sensitized colon, lung and prostate cancer cells to HDACi-induced cell death [42]. Another study reported that the MEK inhibitors PD184352 or AZD6244 in human colon and lung tumor xenograft models enhanced the efficacy of the HDACi MS-275 and suggested this combination as a promising chemotherapeutic strategy [43]. Subsequently, a Phase 1 study was conducted combining the HDACi MS-275 and the MAPK pathway inhibitor sorafenib to treat patients with solid tumors or acute myeloid leukemia (AML) [44]. This combination increased apoptosis in various cancer cell lines and was well tolerated [44]. As a result, combining HDAC and MEK inhibitors was further investigated in other studies [16,18,27,28,42,45].

MEK inhibition alone in pre-clinical studies of GB displayed antitumor effects, but neither enhanced the efficacy of the standard treatment (radiation or TMZ) nor blocked their effectiveness [46]. HDACis, on the other hand, were proposed as promising anticancer compounds capable of targeting GSLCs in single or combination treatments [47]. The current study, therefore, examined the efficacy of combining an HDACi (MS-275) with MEK inhibitors (TAK-733 or trametinib) via a new approach that includes ionizing radiation as an additional strategy against GB. We employed the spheroid culture method for in vitro enrichment of GSLC markers to induce a stem-like phenotype [48,49] and predict the efficacy of the combined treatment against GSLCs.

The Genomics of Drug Sensitivity in Cancer (GDSC) database revealed that the half-maximal inhibitory concentration (IC_50_) of the compounds in GB treatment range from 0.9–51.5 µM for HDACi and 0.1–48.1 µM for MEKi [50]. However, the HDACi and MEKi concentrations were chosen based on the potency of the compounds to reduce the cell viability of the GB-derived spheres at 1 µM alone or in combination with 4 Gy radiation. TMZ, on the other hand, was less effective against the viability of the cells at low concentrations. This was consistent with a study that reported GSLCs to be less sensitive to TMZ treatment via cell viability quantification [51]. Therefore, we decided to use 1 µM HDACi and MEKi, while TMZ was used at a higher concentration of 50 µM.

The combination of the HDAC and MEK inhibitors (MS-275 and TAK-733 or MS-275 and trametinib), both at a low concentration of 1 µM, with 4 Gy radiation decreased the sphere formation rate of the GB-derived spheres. Nevertheless, a higher concentration of TMZ (50 µM) with radiation achieved a similar effect. Overall, given that combination therapy is applied to enhance effectiveness, lower doses of the single compounds involved are often desired to lower the risk of drug toxicity to healthy cells [52]. Hence, the efficacy of the HDAC and MEK inhibitors at very low concentrations suggests an improved safety profile and tolerance level for GB therapy as opposed to TMZ [53].

The expressions of GSLC markers CD44, Nestin and SOX2 required for the maintenance of the GSLC population in GB [3] were used to further measure the effect of the combined therapy. All three GSLC markers are reportedly highly expressed in GB compared to normal brain tissues, indicating that they may be responsible for the progression of the tumor and low patient survival rates [54,55,56]. These GSLC markers were also implicated in GB tumorigenesis and aggressiveness [57]. Therefore, a therapy aimed at downregulating the GSLC markers could be more effective at preventing progression and improving GB treatment [2].

Indeed, we found that combining the HDAC and MEK inhibitors (both at 1 µM) with 4 Gy radiation completely eradicated Nestin and SOX2 protein levels in U251-sph and significantly reduced CD44 protein levels. Similarly, the protein levels of Nestin and SOX2 in U87-sph were also significantly decreased. However, in contrast to U251-sph, the CD44 protein levels were surprisingly upregulated in U87-sph after the combined treatment. This may imply that the sensitivity of CD44 protein levels to the combined HDAC and MEK inhibitor treatment with radiation could be cell line dependent. Similar to our findings, differential responses of protein levels to treatment in the U87-sph and U251-sph were reported [49].

Subsequently, the HDAC and MEK inhibitor treatment with radiation strongly reduced the single positive (CD44+, Nestin+ and SOX2+) populations in both cell lines, except for CD44 expression, which remained unchanged in U87-sph. The differential response of CD44 to the combined treatment in both GB-derived spheres could have also been due to the different genetic alterations present in both cell lines.

Since there has been a dispute regarding whether a single GSLC marker expression accurately identifies the GSLC population [57,58], we further evaluated the double expressions of the GSLC markers after the combined treatment. Again, we found that all three double-positive populations (CD44+Nestin+, Nestin+SOX2+ and CD44+SOX2+) were significantly reduced in both GB-derived spheres by the combined HDAC and MEK inhibitor treatment with radiation. Live–dead staining also revealed that the percentage of the dead cell population in both GB-derived spheres was greatly increased upon the combined treatment with radiation. This finding suggested that the mechanism behind the reduced GSLC marker expression after the combined treatment may be due to the killing of the U251-sph and U87-sph cells. While others have shown that the combination of HDACi and MEKi can enhance tumor cell killing compared to either alone [59], our new approach of including radiation shows a further enhancement of this anti-tumor effect in GB. Although more investigations are required, these data suggested that the combined radiochemotherapy may have great potential against the highly resistant GB cells.

Interestingly, the current standard drug TMZ at a higher concentration (50 µM), alone or with radiation, either did not have any effect or increased the expression of GSLC markers. In addition, the population of dead cells was not significantly changed compared to the sham irradiated control cells. Some studies have reported TMZ chemoresistance of glioma stem cells and GB [60,61]. Another study reported an increase in the CD44+ population in patient-derived GSLCs in response to TMZ and radiation [51]. An explanation for the chemoresistance to TMZ could be an increased expression of a DNA repair enzyme known as MGMT (*O*-6-methylguanine-DNA methyltransferase) [62]. MGMT efficiently repairs the DNA damage caused by TMZ, hence only GB cells with an epigenetically silenced expression of the enzyme can benefit from TMZ treatment [63]. Our data has shown that TMZ alone or with radiation was less effective, whereas the combination of the HDAC and MEK inhibitors with radiation efficiently reduced the GSLC markers.

Some reported anti-tumor mechanisms behind the HDAC and MEK inhibitor combination in other cancer studies include enhanced production of ROS (reactive oxygen species) [42], activation of cell-cycle inhibitors [45], increased apoptosis and deregulated survival pathways [59]. In addition to this, we showed that by including the cytotoxic effects of radiation as a novelty, the combined radiochemotherapy treatment could be more effective in the killing of GB tumor cells.

A limitation to our study was that the GB-derived spheres did not fully represent the GSLC population due to the artificial culture conditions, nor did our work establish the combination index of the HDAC and MEK inhibitors. To better elucidate the efficacy of the combined therapy with radiation, further research is necessary using experimental models that involve purely isolated GSLC populations. This can be achieved through fluorescence-activated cell sorting (FACS) to isolate side populations expressing GSLC markers [64] or low-passage patient-derived primary GSLCs [65]. In addition, in vivo validations are required since the in vitro cultures fail to either address the heterogeneity of the original tumor or recapitulate the hierarchy of GSLCs [3,66].

## 5. Conclusions

In conclusion, our findings support the proposition that a combination therapy approach against GB may be an effective way to improve outcomes [40]. The efficacy of this approach was demonstrated by a reduction in the ability of the GB-derived spheres to form spheroids after the combined HDAC and MEK inhibitor (MS-275 and TAK-733 or MS-275 and trametinib) treatment with radiation. The combined treatment with radiation further decreased the expression of the GSLC markers Nestin and SOX2, while the effect on CD44 was cell line dependent. Moreover, the combined treatment was more efficient compared to the standard treatment of TMZ and radiation. Although more research is needed to validate the efficacy of this combination strategy, the results suggested that this may be a promising multimodal therapy against the highly resistant GB that can inhibit recurrence and increase the survival of patients.

## Data Availability

Data is contained within the article or supplementary material.

## Supplementary Materials

The following are available online at https://www.mdpi.com/article/10.3390/cells11050775/s1. Figure S1: Activities of the HDACi MS-275 (MS) and the MEKi TAK-733 (TAK) or trametinib (TRA) in GSLC lines. (**a**) Western blots showing the increased acetylation effect of MS-275 on histone H3 in whole-cell lysates from U87-sph and U251-sph cells (*n* = 2). (**b**) Western blots showing the inhibitory effects of TAK-733 and trametinib on phosphorylated MAPK (pMAPK) in U87-sph and U251-sph cells. Figure S2: Cell viability decreased with increasing concentration of compounds (MS-275, TAK-733, trametinib and TMZ), with and without 4 Gy radiation. (**a**) Cell viability of U87-sph after the 1 µM, (**b**) 10 µM and (**c**) 50 µM compound and radiation treatments. (**d**) Cell viability of U251-sph after the 1 µM, (**e**) 10 µM and (**f**) 50 µM compound and radiation treatment. Values were normalized to control samples (DMSO) set to 1. Bars not visible represent values less than or equal to 0. Data represent the mean ± SEM of three independent experiments performed in quadruplicates (*t*-test, *** *p* ≥ 0.0001). Figure S3: Treatment of HDAC and MEK inhibitors with radiation-inhibited sphere formation of GSLC lines. (**a**) Representative images of spheres formed 14 days after treatment with compounds and radiation in U87-sph cells and (**b**) U251-sph cells (scale bar: 100 µm). Figure S4: Gating strategy and FMO controls for the flow cytometry results shown in Figure 5 and Figure 6. (**a**) Cells were gated based on size and granularity using side scatter area (SSC-A) vs. forward scatter area (FSC-A) to remove debris and clumped cells. From this cell gate, single cells (singlets) were sub-gated using forward scatter height (FSC-H) vs. FSC-A to remove doublets. From the singlets gates, the control and treated samples were sub-gated to obtain the negative and positive single or double populations. (**b**) Fluorescence minus one (FMO) controls used to gate the double-positive populations for Figure 6 are shown. The gating region on the FMO controls was set to contain less than 1% of the cells for double-positive populations. FMO control minus (−) of each indicated antibody is shown. Figure S5: Representative pictures for the single positive populations of (**a**) CD44+ and (**b**) SOX2+ in U251-sph for Figure 5b. Unstained, FMO and isotype control (SOX2) for gating positive population are shown. Figure S6: Representative pictures for the double-positive populations of (**a**) Nestin+SOX2+ and (**b**) CD44+SOX2+ in U251-sph for Figure 6b. Figure S7: Representative pictures for the single positive populations of (**a**) CD44+, (**b**) SOX2+ and (**c**) Nestin+ in U87-sph for Figure 5c. Unstained, FMO and isotype control (SOX2) for gating positive populations are shown. Values inside each flow cytometric plot represent the percentage of single positive cells from a total of approximately 2 × 10^4^ cells acquired. Figure S8: Representative pictures for the double-positive populations of (**a**) CD44+Nestin+, (**b**) Nestin+SOX2+ and (**c**) CD44+SOX2+ in U87-sph for Figure 6c.
