# Supplementary material for: Combining HDAC and MEK Inhibitors with Radiation against Glioblastoma-Derived Spheres
Source: Cells. 2022 Feb 23;11(5):775. doi: 10.3390/cells11050775 (PMC8909581; doi:10.3390/cells11050775)
Supplement: Supplementary file 1 [file cells-11-00775-s001.zip › cells-1600700-supplementary.pdf]

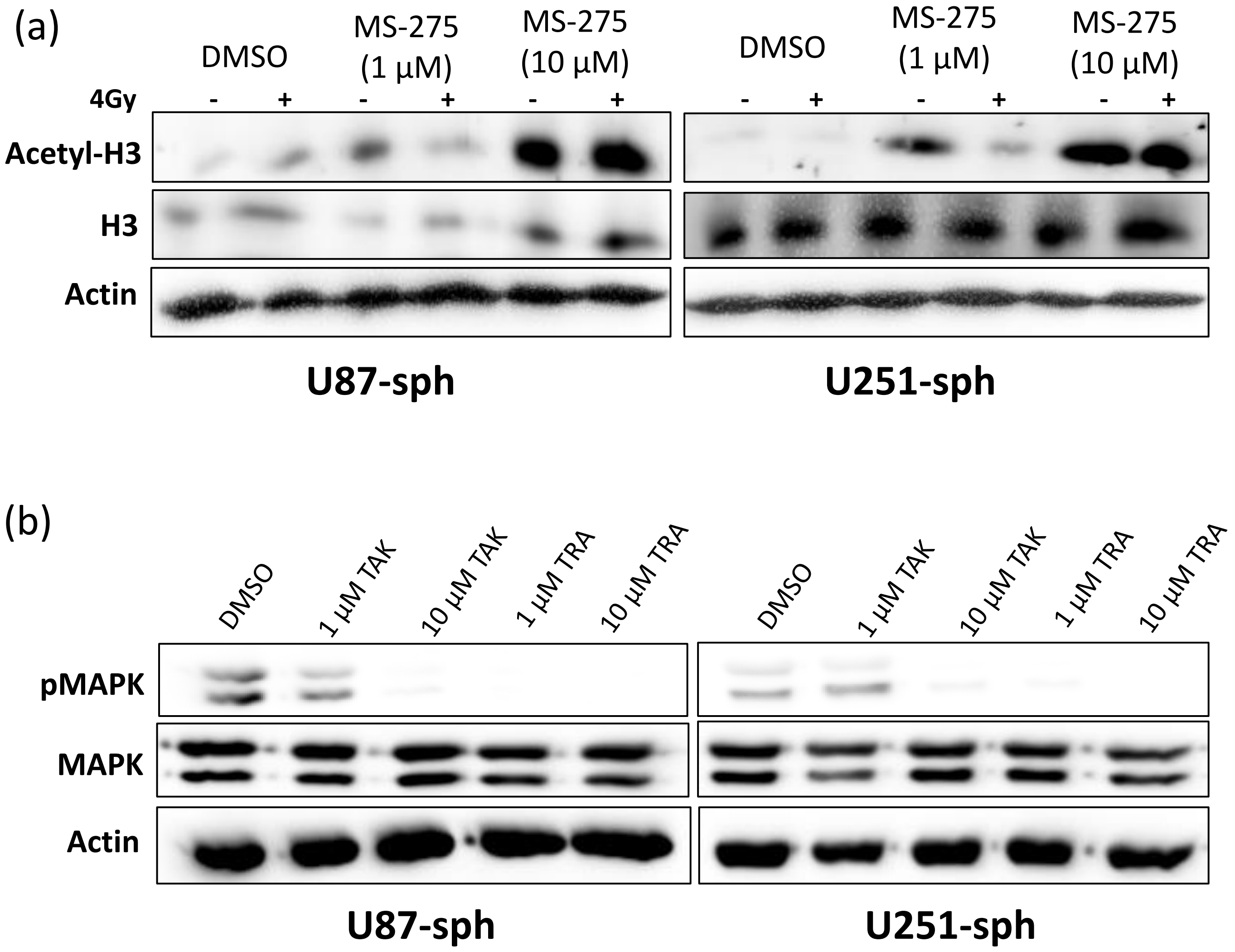

Figure S1: **Activities of the HDACi MS-275 (MS) and the MEKi TAK-733 (TAK) or trametinib (TRA) in GSLC lines.** (a) western blots showing the increased acetylation effect of MS-275 on Histone H3 in whole cell lysates from U87-sph and U251-sph cells (n=2) (b) western blots showing the inhibitory effects of TAK-733 and trametinib on phosphorylated MAPK (pMAPK) in U87-sph and U251-sph cells.

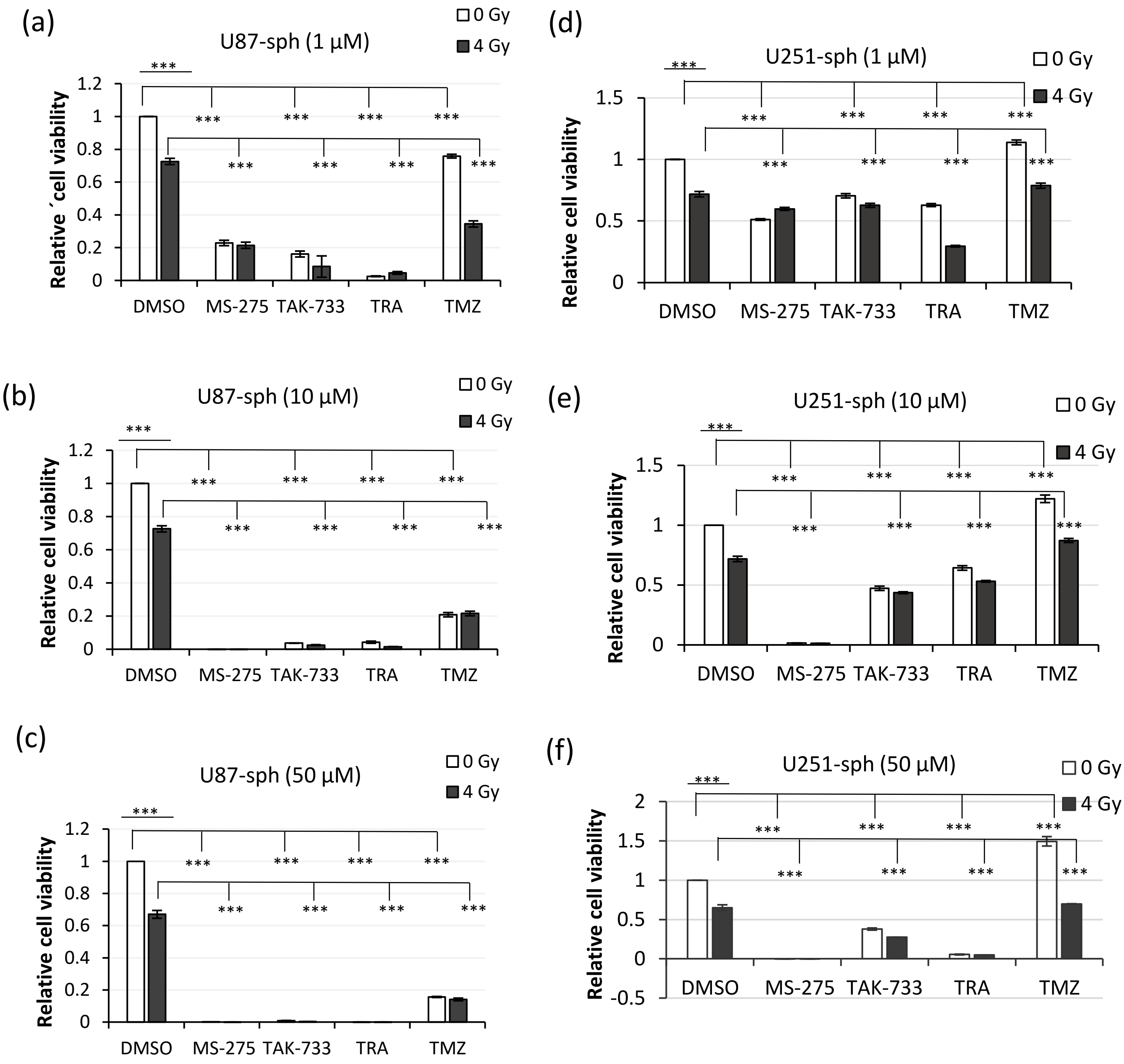

Figure S2: **Cell viability decreased with increasing concentration of compounds (MS-275, TAK-733, Trametinib and TMZ) with and without 4 Gy radiation** (a) Cell viability of U87-sph after 1 µM (b) 10 µM and (c) 50 µM compound and radiation treatment (d) Cell viability of U251-sph after 1 µM (e) 10 µM and (f) 50 µM compound and radiation treatment. Values were normalized to control samples (DMSO) set to 1. Bars not visible represent values less than or equal to 0. Data represents mean ± SEM of three independent experiments performed in quadruplicates (t-test ***$p \geq 0.0001$).

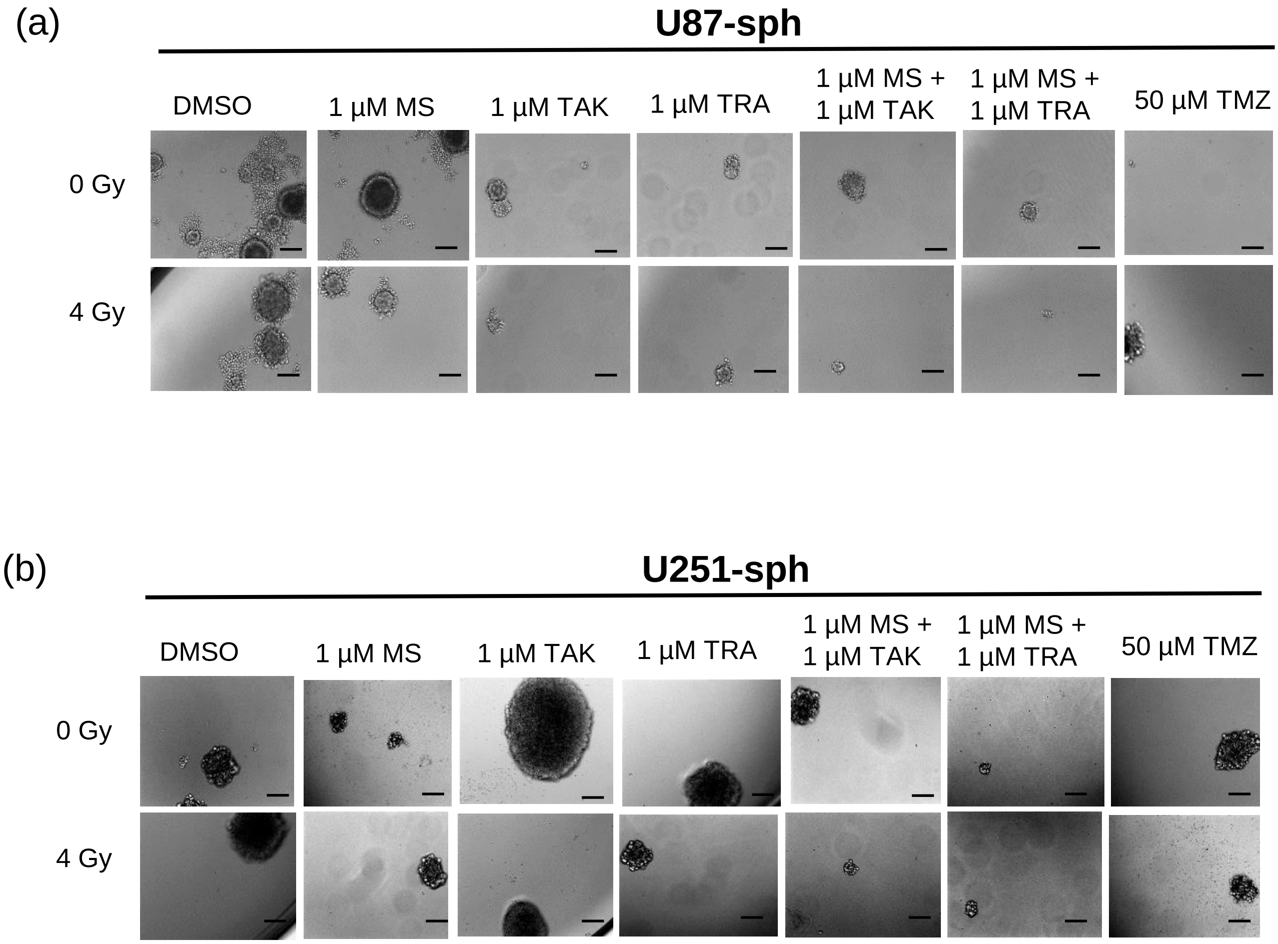

Figure S3: **Treatment of HDAC and MEK inhibitor with radiation inhibited sphere formation of GSLC lines** (a) Representative images of spheres formed 14 days after treatment of compounds and radiation in U87-sph cells and (b) U251-sph cells (scale bar, 100 µm).

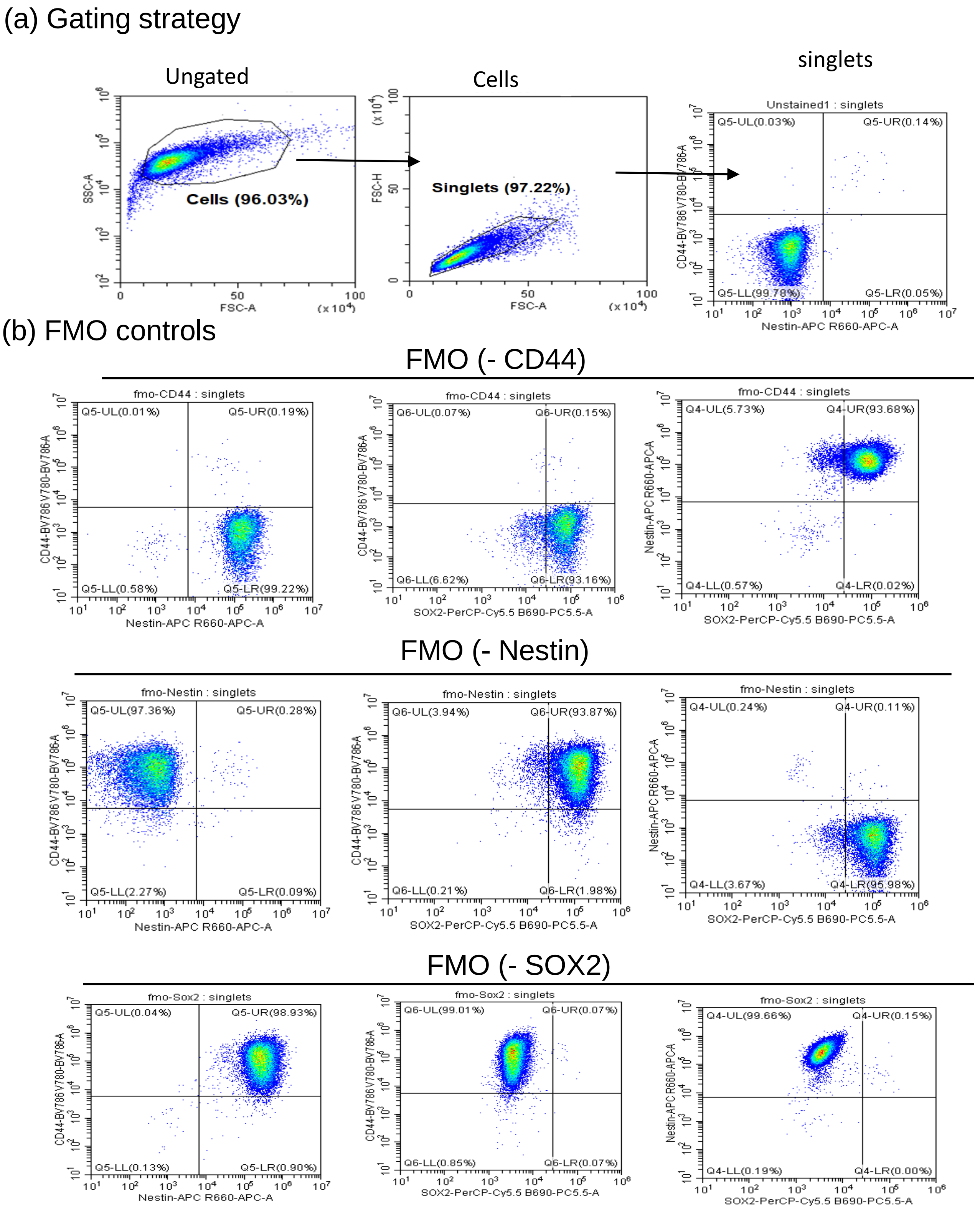

Figure S4: **Gating strategy and FMO controls for the flow cytometry results shown in Figure 5 and 6.** (a) Cells were gated based on size and granularity using side scatter area (SSC-A) vs forward scatter area (FSC-A) to remove debris and clumped cells. From this cell gate, single cells (singlets) were sub-gated using forward scatter height (FSC-H) vs FSC-A to remove doublets. From the singlets gates, the control and treated samples were sub-gated to obtain the negative and positive single or double populations. (b) Flourescence minus one (FMO) controls used to gate the double positive populations for Figure 6 are shown. The gating region on the FMO controls were set to contain less than 1% of the cells for double positive populations. FMO controls minus (-) the indicated antibodies are shown.

U251-sph

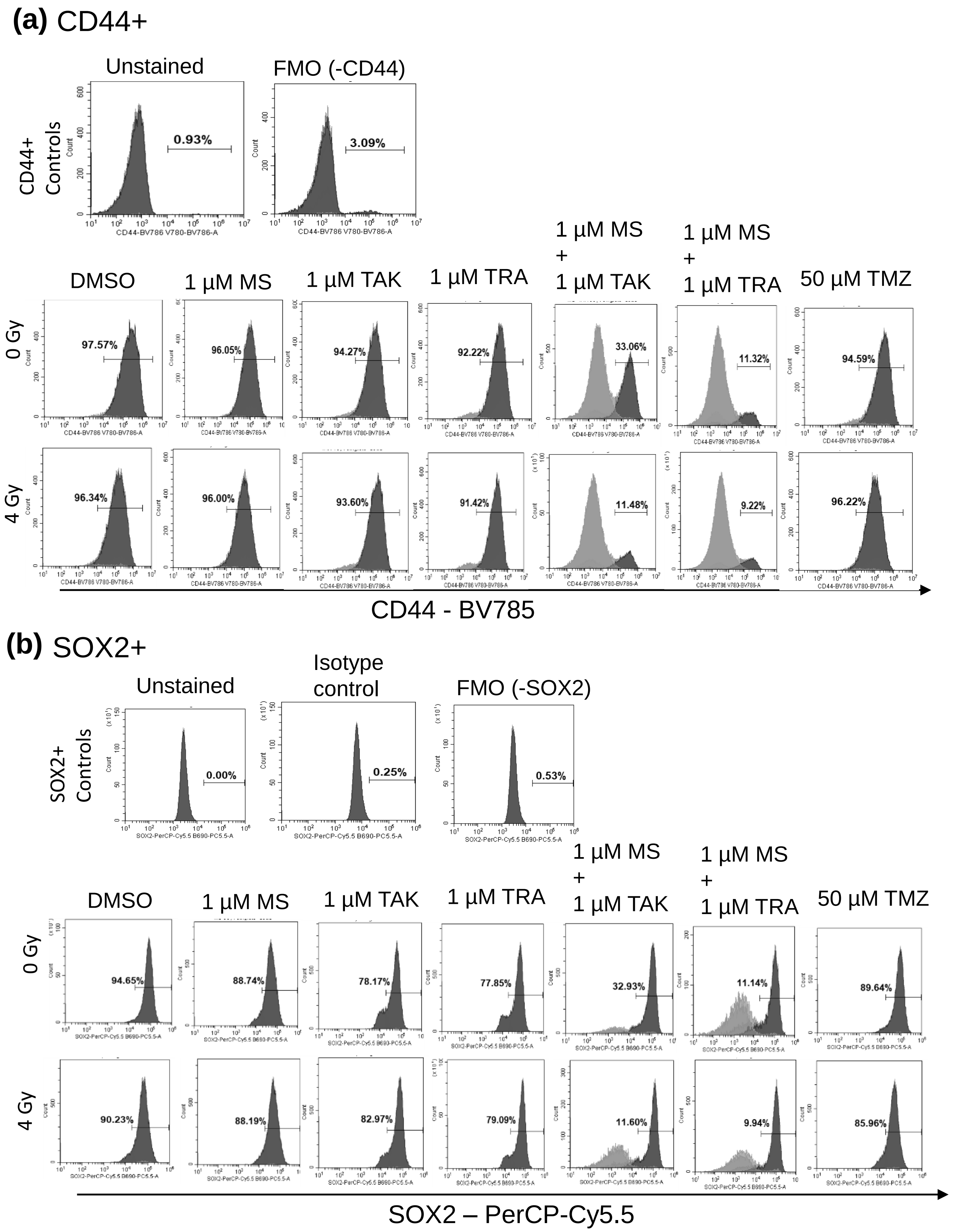

Figure S5: Representative pictures for the single positive populations of (a) CD44+ (b) SOX2+ in U251-sph for Figure 5b. Unstained, FMO and isotype control (SOX2) for gating positive population are shown.

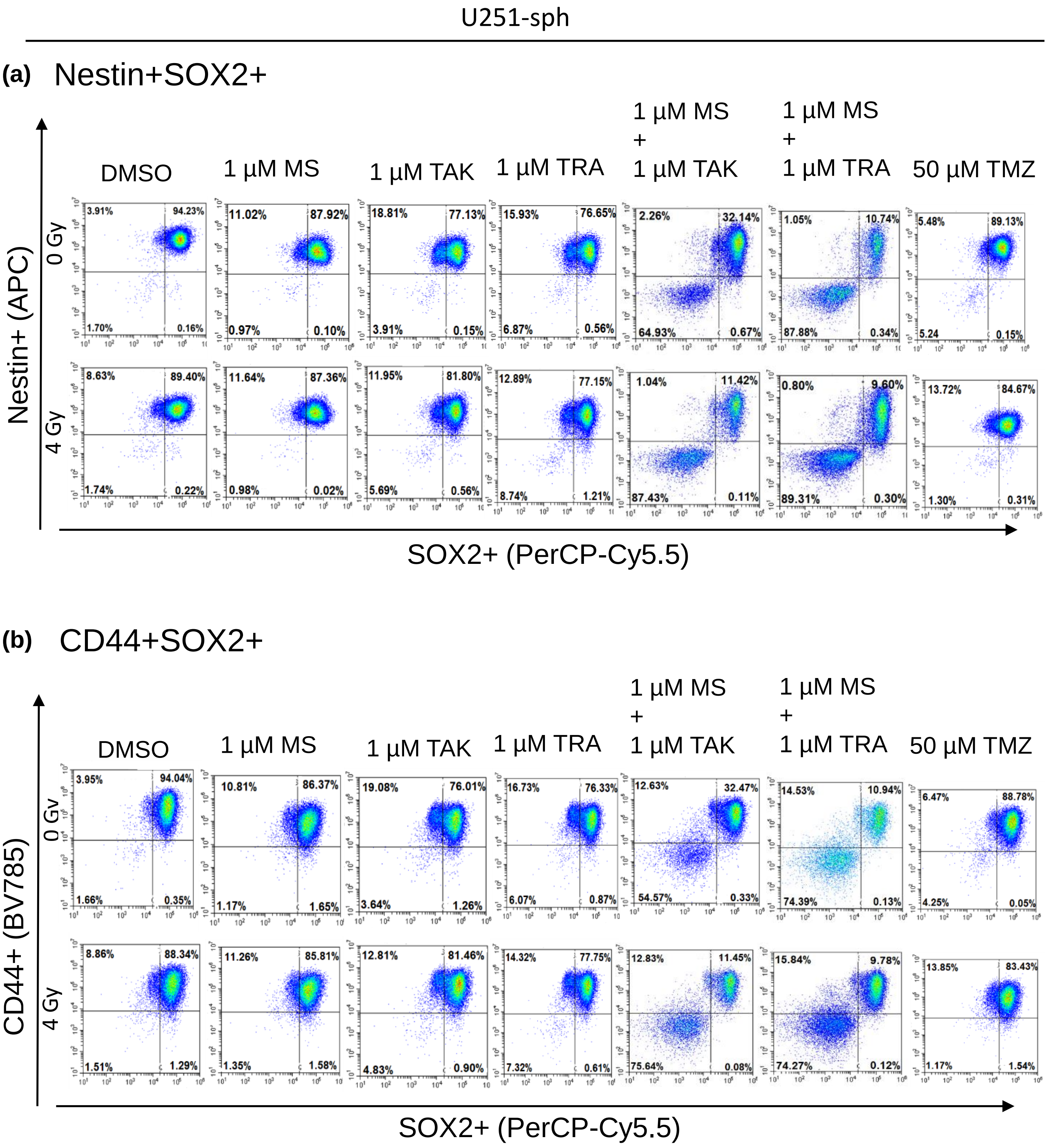

Figure S6: Representative pictures for the double positive populations of (a) Nestin+SOX2+ (b) and CD44+SOX2+ in U251-sph for Figure 6b.

U87-sph

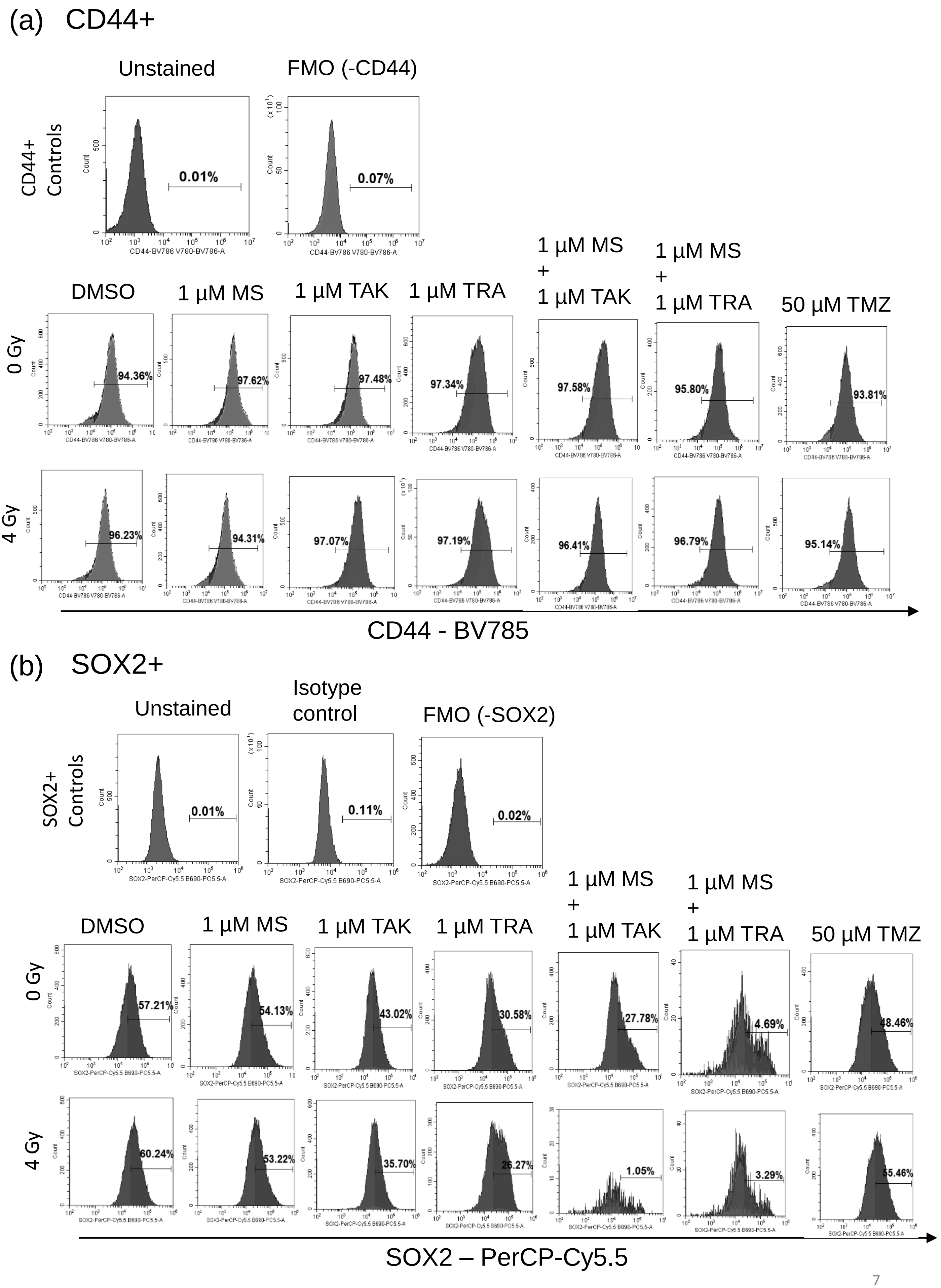

Figure S7 *cont.*

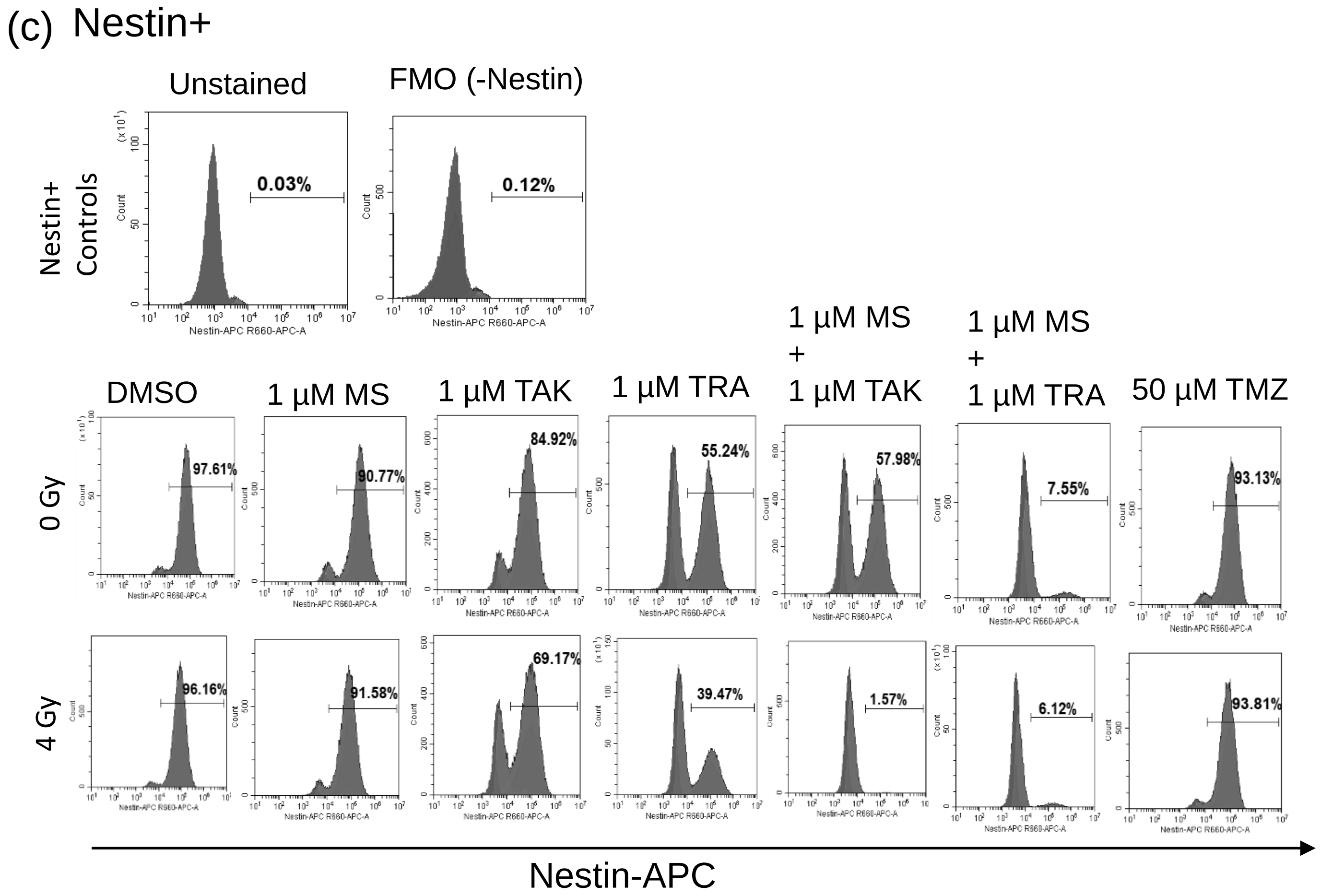

Figure S7: Representative pictures for the single positive populations of (a) CD44+ (b) SOX2+ (c) and Nestin+ in U87-sph for Figure 5c. Unstained, FMO and isotype control (SOX2) for gating positive populations are shown. Values inside each flow cytometric plot represents percentage of single positive cells from a total of approximately 2 x 10^4 cells acquired.

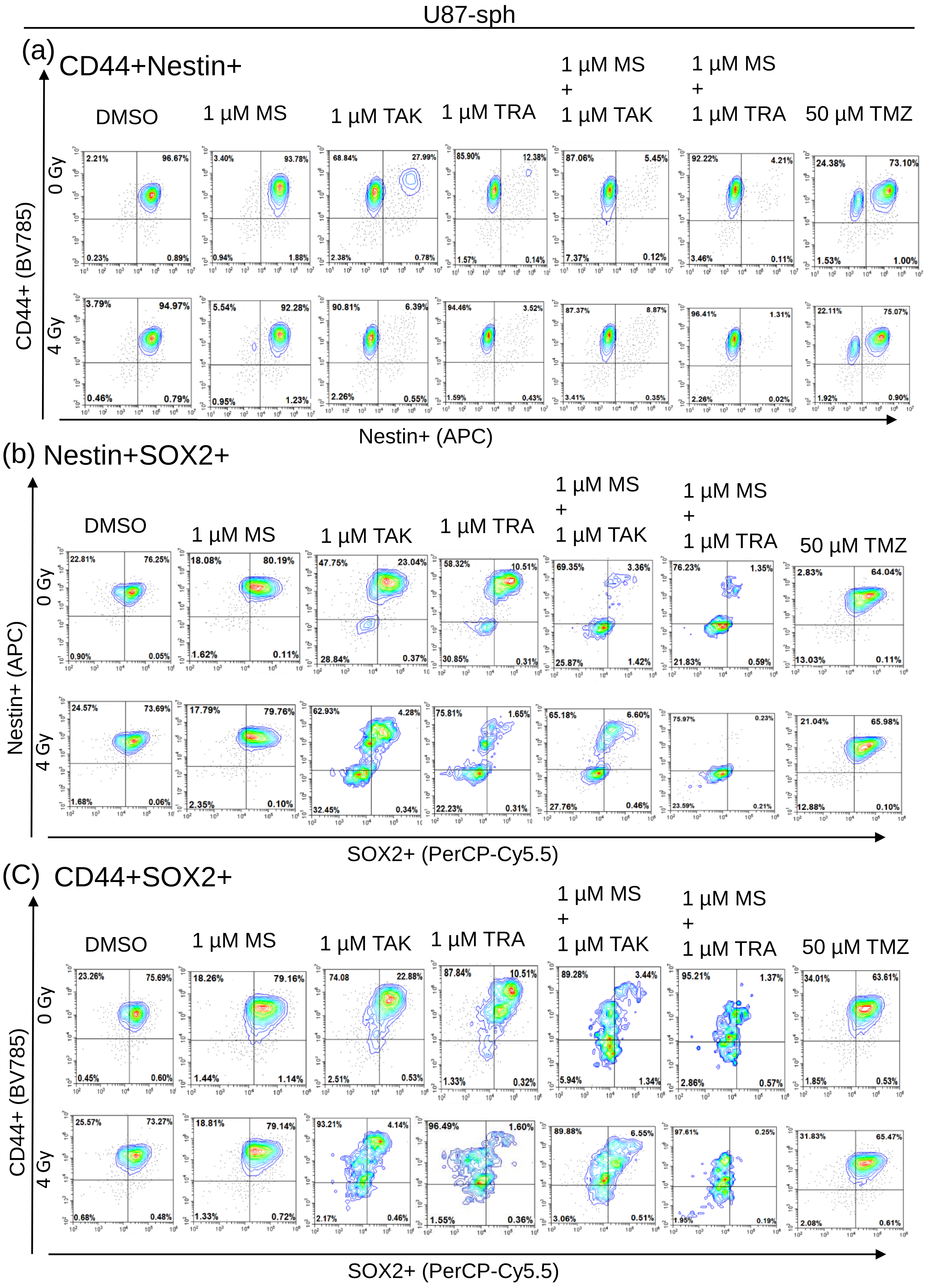

Figure S8: Representative pictures for the double positive populations of (a) CD44+Nestin+ (b) Nestin+SOX2+ (c) and CD44+SOX2+ in U87-sph for Figure 6c.
